# Correction to “Segmentectomy versus lobectomy for small‐sized pure solid non‐small cell lung cancer”

**DOI:** 10.1111/1759-7714.15292

**Published:** 2024-03-20

Li Z , Xu W , Pan X , Wu W , Chen L . Segmentectomy versus lobectomy for small‐sized pure solid non–small cell lung cancer. Thorac Cancer. 2023;14(11):1021–1028. 10.1111/1759-7714.14840 36882365 PMC10101834

In the author list and the “Correspondence” on the title page, the name of the corresponding author “Lian Chen” was incorrect. Besides, the name “Lian Chen” was also incorrect in the “ORCID” section on page 7 (before the “REFERENCES”). The correct name should be “Liang Chen”.

We apologize for this error.

